# Fas-Activated Serine/Threonine Kinase Governs Cardiac Mitochondrial Complex I Functional Integrity in Ischemia/Reperfusion Heart

**DOI:** 10.3389/fcell.2020.630421

**Published:** 2021-01-28

**Authors:** Xiyao Chen, Guangyu Hu, Yuanyuan Wang, Congye Li, Fuyang Zhang

**Affiliations:** ^1^Department of Geriatrics, Xijing Hospital, Air Force Medical University, Xi'an, China; ^2^Department of Cardiology, Xijing Hospital, Air Force Medical University, Xi'an, China; ^3^Department of Neurology, Xijing Hospital, Air Force Medical University, Xi'an, China; ^4^Department of Physiology and Pathophysiology, Basic Medicine School, Air Force Medical University, Xi'an, China

**Keywords:** FASTK, mitochondrion, complex I, ischemia/reperfusion, MTND6

## Abstract

Cardiac energy homeostasis is strictly controlled by the mitochondrial complex-mediated respiration. In the heart, mitochondrial complex I is highly susceptible to functional and structural destroy after ischemia/reperfusion (I/R), thereby contributing to myocardial energy insufficiency and cardiomyocyte death. Fas-activated serine/threonine kinase (FASTK) is recently recognized as a key modulator of mitochondrial gene expression and respiration. However, the role of FASTK in cardiac I/R process is undetermined. Here, we show that FASTK expression was down-regulated in the post-I/R heart. The reactive oxygen species scavenger N-acetyl-L-cysteine reversed I/R-induced FASTK down-regulation. Genetic deletion of FASTK exacerbated I/R-induced cardiac dysfunction, enlarged myocardial infarct size, and increased cardiomyocyte apoptosis. Compared with the wild type control, the FASTK deficient heart exhibited a lower mRNA expression of NADH dehydrogenase subunit-6 (MTND6, a mitochondrial gene encoding a subunit of complex I) and was more vulnerable to I/R-associated complex I inactivation. Replenishment of FASTK expression via adenovirus-mediated gene delivery restored mitochondrial complex I activity and ameliorated cardiomyocyte death induced by I/R, whereas these beneficial effects were blocked by the co-treatment with rotenone, a specific complex I inhibitor. *in vivo* experiments further confirmed that cardiac overexpression of FASTK ameliorated I/R-related MTND6 down-regulation and mitochondrial complex I inactivation, thereby protecting the heart against I/R injury. Collectively, these data for the first time identify that the down-regulation of FASTK is a direct culprit behind the loss of mitochondrial complex I functional integrity and cardiac injury induced by I/R process. Targeting FASTK might be a promising and effective strategy for MI/R intervention.

## Introduction

Coronary artery disease is a pandemic that poses a major threat to the public health in both developing and developed countries (Joseph et al., 2017). In patients with coronary artery disease, acute myocardial infarction (MI) is a leading cause of death and the best strategy for the clinical management of MI is to restore coronary blood flow by the thrombolytic approach or percutaneous coronary intervention (Reed et al., 2017). However, when the obstructed coronary artery is re-opened, the ischemic myocardium is further damaged by a sudden increase of blood supply, a process named as myocardial ischemia/reperfusion (I/R) injury (Heusch and Gersh, 2017). Currently, there is no effective therapy for preventing myocardial I/R injury. In this respect, myocardial I/R injury remains a neglected issue for cardioprotection in patients receiving revascularization therapies (Hausenloy and Yellon, 2013). Thus, it is extremely urgent to develop more effective and safe treatment for cardiac I/R damage.

The heart is a high energy-consuming organ and largely relies on the energy supplied by cardiac mitochondria (Bonora et al., 2019). It is evident that the cardiac mitochondrion plays a key role in the regulation of myocardial I/R injury (Kuznetsov et al., 2019). Mitochondrial complex I (also known as NADH ubiquinone oxidoreductase) is a large, multi-subunit, integral membrane protein complex that catalyzes the first step of electron transfer chain (ETC) (Formosa et al., 2018). Mitochondrial complex I activity is essential for maintaining functional integrity of the respiratory complexes and to assure efficient transfer of electrons between ETC complexes (Karamanlidis et al., 2013). In response to I/R, cardiac mitochondrial complex I is highly susceptible to functional and structural damage, which contributes to I/R-associated mitochondrial ETC disorder, respiratory dysfunction, and energy supply insufficiency (Kang et al., 2018). The loss of cardiac mitochondria-derived energy substrates directly causes contractile dysfunction, redox imbalance, and even cardiomyocyte death in response to I/R (Wüst et al., 2016; Hou et al., 2019). These findings suggest that the amelioration of mitochondrial complex I dysfunction is a promising strategy for the treatment of myocardial I/R injury. However, the mechanisms underlying I/R-associated mitochondrial complex I dysfunction remain elusive and effective therapies are still lacking.

Fas-activated serine/threonine kinase (FASTK) is a mitochondrion-localized protein that is widely expressed in various tissues such as the heart, liver, kidney, lung, and skeletal muscle (Jourdain et al., 2017). Recently, FASTK has been identified as a key RNA-binding protein involved in the alternative RNA splicing (Simarro et al., 2007). In mammalian cells, FASTK specifically interacts with the mRNA of NADH dehydrogenase subunit 6 (MTND6, a mitochondrial gene encoding a subunit of complex I) at various sites and is essential for the processing and maturation of MTND6 mRNA (Jourdain et al., 2015). *In vivo* and *in vitro* studies have confirmed that genetic ablation of FASTK decreased the expression of MTND6 and thereafter suppressed mitochondrial complex I activity approximately by 50% (García Del Río et al., 2018; Gomez-Niño et al., 2018). These data reveal that FASTK is a novel and important modulator of mitochondrial complex I activity and respiratory function. However, the role of FASTK in the regulation of cardiovascular physiology and pathophysiology is totally unknown, especially in the context of myocardial I/R process. Therefore, the present study aims to determine whether FASTK influences myocardial I/R injury and to clarify the involvement of FASTK in the regulation of cardiac mitochondrial homeostasis.

## Materials and Methods

### Animals and Myocardial I/R Model

All animal study protocols were adhered to the National Institutes of Health Guidelines for the Use of Laboratory Animals and were approved by the Air Force Medical University Committee on Animal Care. Transgenic mice with FASTK knockout (KO) were obtained from the Jackson Laboratory and were maintained and genotyped as we previously described (Zhang et al., 2020). Mice were housed in a temperature-controlled environment (23 ± 2°C) with a 12/12 h light/dark cycle. Myocardial I/R models were established in age-matched KO mice and their wild-type (WT) littermates as we previously described (Li et al., 2020). Briefly, mice were anesthetized with 1–2% isoflurane and myocardial ischemia was induced by the ligation of the left anterior descending coronary artery by a 6-0 silk suture slipknot. After 40 min of myocardial ischemia, the slipknot was released and the myocardium was reperfused. Sham-operated mice (Sham) underwent the same surgical procedures except that the artery ligation. Mice were sacrificed by cutting the carotid artery after 3 h (for measuring FASTK mRNA and protein and measuring cardiomyocyte apoptosis) and 24 h (for echocardiography and Evans/TTC staining) post-reperfusion.

### Echocardiography

Mice were subjected to 1–2% isoflurane anesthesia and M-mode echocardiography was performed using a Visualsonics 770 echocardiography system as we previously described (Li et al., 2020). Hearts were viewed in the short-axis between the two papillary muscles and each measurement was obtained using M-mode by the average results from three consecutive heart beats. Ejection fraction (EF) and fraction shortening (FS) were calculated.

### Terminal Deoxyribonucleotidyl Transferase Dutp Nick End Labeling Staining

Cardiomyocyte apoptosis was determined by the TUNEL kit (Roche, Germany) according to the manufacturer's instructions. The percentage of apoptotic nuclei was calculated by counting the number of TUNEL-positive cardiomyocyte nuclei normalized by the total number of DAPI-positive nuclei. Apoptosis was evaluated in six randomized fields per tissue section of the ischemic zone.

### Evans Blue/Triphenyltetrazolium Chloride Double-Staining

The infarct size and the area-at-risk (AAR) were defined by Evans blue/TTC double staining as we previously described (Li et al., 2020). In brief, 1% Evans blue in saline (0.2 mL) was injected retrograde into the brachiocephalic artery. The non-ischemic area, which was not at risk, was stained blue. The heart was excised and cut into five 1-mm-thick transverse slices, parallel to the atrioventricular groove. AAR was calculated by dividing the total non-blue area by the total area of the left ventricle sections. Each slice was incubated in 1% TTC solution at 37°C for 15 min to differentiate infarct area (pale) from viable (red) myocardium tissues. The AAR and infarct size from each section were both measured using the Image J software.

### Neonatal Mouse Ventricular Myocyte Isolation

NMVMs from 1- to 2-day-old WT or KO mouse pups were isolated and cultured as we previously described (Chen et al., 2017). Immediately after euthanasia of mouse pups, hearts were removed and ventricles were minced. Thereafter, NMVMs were isolated with 1.0 mg/mL collagenase type II (Thermo, USA). NMVMs were re-suspended in high glucose DMEM (Sigma, USA) containing 10% fetal bovine serum (FBS), 10 mM HEPES, and 0.1 mM 5-Bromo-2′-deoxyuridine (BrdU, Sigma, USA), and plated in culture dishes for 90 min to allow attachment of fast-adherent fibroblasts. Non-adherent cells were collected and seeded into the culture plate. On the next day, the culture medium was replaced with M199 containing 10% FBS, 10 mM HEPES, and no BrdU.

### *In vitro* Simulated I/R Model

NMVMs were exposed to hypoxia/re-oxygenation to simulate *in vivo* myocardial I/R process as we previously described (Chen et al., 2017). The medium was replaced with DMEM lacking FBS before the cells were placed into a hypoxic incubator (95% N_2_ and 5% CO_2_ at 37°C). After 6 h in the hypoxic incubator, cells were replaced with normal M199 medium with 10% FBS and transferred to a normal incubator (95% O_2_ and 5% CO_2_ at 37°C) for another 2 h to reoxygenate.

### Myocardial Caspase Activity Measurement

Caspase-3 and−9 activity were measured by the commercial kits (Abcam, USA) according to the manufacturer's instruction described and normalized to protein concentrations of the samples.

### Mitochondrial Complex Activity Measurement

Mitochondrial fractions were subjected to measurements of complex I and II+III activity using the commercial kits (Abcam, USA) as the manual described. Briefly, freshly isolated mitochondrial fractions were suspended in phosphate buffered saline with 10% detergent. Protein concentrations of mitochondrial components were measured. 25 μg (for complex I) or 100 μg (For complex II+III) mitochondrial protein component was added for the reaction. Enzyme activities were measured spectrophotometrically.

### Plasma Lactate Dehydrogenase Activity Measurement

Plasma samples were harvested from mice 3 h after the reperfusion of mice subjected to sham or myocardial I/R operation. The enzyme activity of LDH was spectrophotometrically assayed using a LDH activity assay kit (Beyotime, China).

### Intra-Myocardial Adenovirus Injection

Adenovirus vectors carrying full length FASTK gene (Ad-FASTK) or control vectors (Ad-GFP) were delivered into the heart as we previously described (Li et al., 2020). Briefly, mice were anesthetized by inhaling 2% isoflurane and the heart was smoothly “popped out” through a small hole at the 4th intercostal space. Adenovirus vectors were diluted to 2.5 × 10^11^ particles in 25 μL saline and then injected into left ventricular (LV) free wall by a Hamilton syringe with a needle size of 30.5. Intramyocardial injections were performed: (1) starting from apex and moving toward to the base in LV anterior wall; (2) at the upper part of LV anterior wall; and (3) starting at the apex and moving toward to base in LV posterior wall. After adenovirus delivery, the heart was immediately placed back into the chest, carefully closure of muscle, and the skin suture. Mice received sham or I/R operation at the 7th day post-injection.

### Real-Time PCR

Total RNA was isolated from heart tissues or cardiomyocytes using the MiniBEST universal RNA extraction kit (Takara, China). Total RNA was then reversely transcribed into cDNA using the PrimeScript™ RT reagent kit with gDNA eraser (Takara, China). RT-PCR was performed in triplicate using a SyBR RT-PCR detection kit (Cowin, China) and a CFX96 system (Bio-Rad, USA). Primer sequences are shown as below: FASTK forward (5′-3′): GGGGAGTCATGGTCTCCAC; FASTK reverse (5′-3′): CTTGCTGGGTCCCAAACAA; MTND6 forward (5′-3′): CGGTAATACGACTCACTATAGGGAGACCCGCAAACAAAGATCACCC; MTND6 reverse (5′-3′): TGGGTTAGCATTAAAGCCTTCAC; β-actin forward (5′-3′): GGCTGTATTCCCCTCCATCG; β-actin reverse (5′-3′): CCAGTTGGTAACAATGCCATGT.

### Western Blot

Cell lysates from *in vitro* and *in vivo* samples were collected, separated by SDS-PAGE, and transferred into PVDF membranes. The membranes were incubated using the primary antibody. The primary antibodies used included FASTK (1:500, Santa Cruz, USA) and β-actin (1:1,000, Santa Cruz, USA). After washing by PBS with 0.1% tween, membranes were incubated at room temperature for 1 h with horseradish peroxidase (HRP)-conjugated goat anti-mouse or goat anti-rabbit secondary anti-bodies (1:5,000, Cowin, China). Proteins were visualized using an enhanced chemiluminescence kit (Merck Millipore, USA). The film was scanned by the ChemiDocXRS equipment (Bio-Rad, USA), and densities of the bands were analyzed by the Quantity One software (Bio-Rad, USA).

### Cardiac ROS Detection

The heart was freshly and carefully cut and the infarcted left ventricular anterior wall was isolated. The myocardial tissue was cut into pieces and prepared into a single cell suspension. After incubating for 30 min at 37°C with DCFH-DA probe (10 nmol/L), the fluorescence intensity was measured with a microplate reader at 488/525 nm (excitation/emission wavelength).

### Statistical Analysis

Statistical analyses were conducted with the Graphpad Prism 8.0 software. Data are expressed as mean ± standard deviation (SD) for the indicated number of experiments or mice. The difference between two groups was compared by an unpaired student's *t*-test (two-tailed). For more than two group, one-way or two-way ANOVA was used and followed by a Turkey test. A *P*-values < 0.05 was considered statistically significant.

## Results

### FASTK Expression Was Declined Due to ROS Burst in I/R Heart

**Figure 1:**
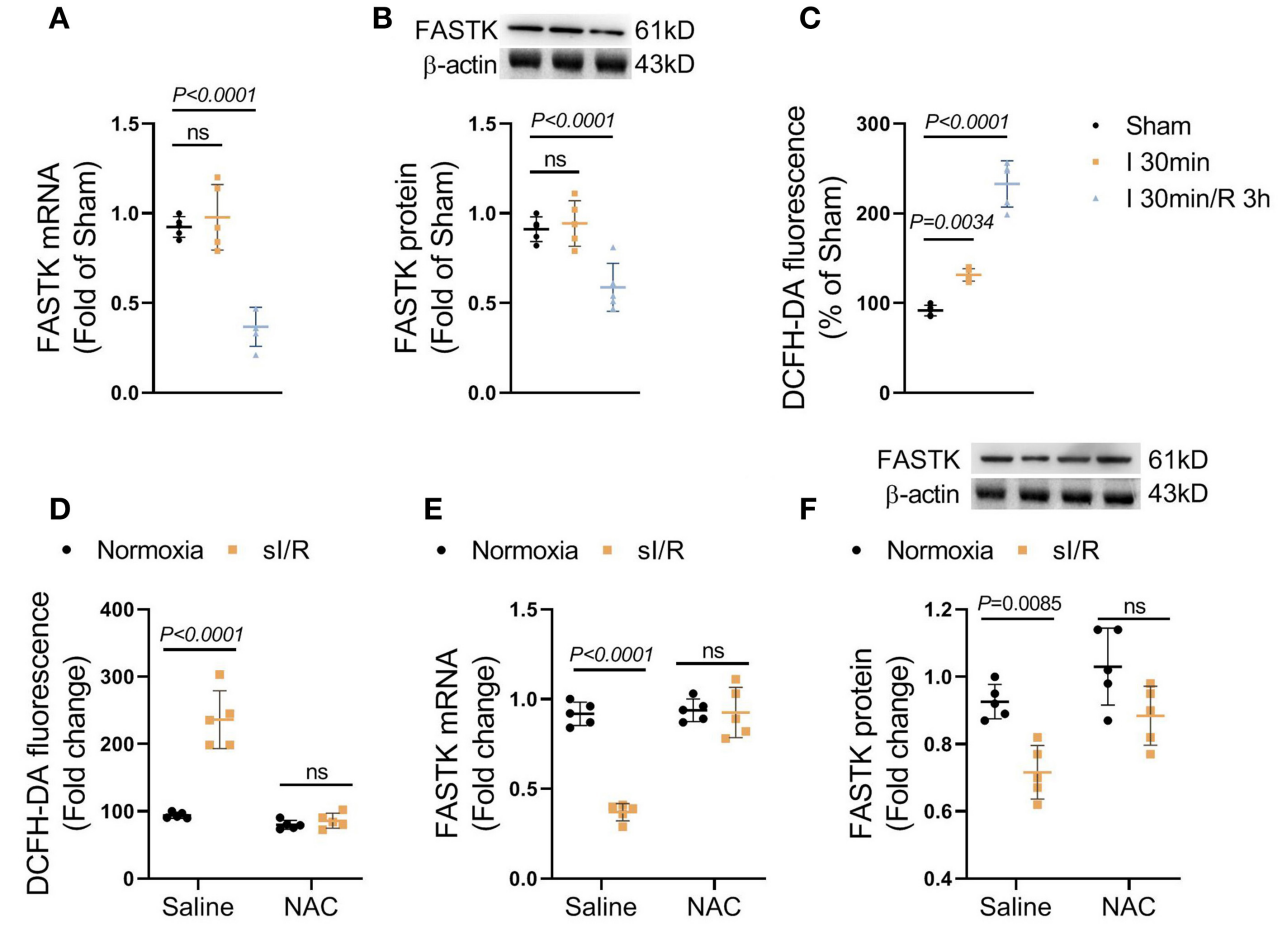
FASTK expression was repressed in post-I/R heart. Male C57BL6J mice were randomly received sham, 40 min myocardial ischemia, or 3 h reperfusion following 40 min ischemia. **(A)** The mRNA levels of FASTK in left ventricular tissues were determined by RT-PCR and normalized to β-actin mRNA expression. **(B)** The protein levels of FASTK were determined by Western blot and normalized to β-actin protein expression. **(C)** Fresh heart tissues were stained by a ROS probe DCFH-DA and the relative fluorescence intensity was calculated. Primary NMVMs were isolated and subjected to sI/R by hypoxia/reoxygenation, with or without NAC (10 mM) co-treatment. **(D)** 1 × 10^6^ NMVMs were stained by DCFH-DA and the relative fluorescence intensity was measured. **(E)** The mRNA levels of FASTK were determined by RT-PCR and normalized to β-actin mRNA expression. **(F)** The protein levels of FASTK were determined by Western blot and normalized to β-actin expression. Data are shown as mean ± SD and analyzed by one-way ANOVA, followed by a Bonferroni *post-hoc* test. I/R, ischemia/reperfusion; ROS, reactive oxygen species; NMVMs, neonatal mouse ventricular myocytes; NAC, N-acetyl-L-cysteine.

To explore the role of FASTK in myocardial I/R process, we first observed the expression change of FASTK in post-I/R heart tissues. We found that the mRNA and protein expression levels of FASTK were unchanged in response to 40 min of ischemia (Figures 1A,B). However, the mRNA and protein levels of FASTK robustly declined in response to the following 3 h reperfusion (Figures 1A,B). These data suggest that cardiac FASTK expression was suppressed by the process of I/R but not the ischemic insult. As expected, reperfusion induced a significant production of ROS as evidenced by the increased fluorescence of DCFH-DA (Figure 1C). Consistently, *in vitro* simulated I/R (sI/R) also induced a significant ROS accumulation in NMVMs (Figure 1D). The mRNA and protein levels of FASTK markedly decreased in cardiomyocytes exposed to simulated I/R (sI/R), consistent with the *in vivo* observations (Figures 1E,F). However, the elimination of ROS by NAC (a potent ROS scavenger) totally blocked sI/R-induced FASTK down-regulation (Figures 1E,F). These data for the first time reveal that cardiac FASTK expression is repressed by I/R due to the burst of intracellular ROS and suggest that the down-regulation of FASTK might involve in the regulation of myocardial I/R injury.

### Genetic Ablation of FASTK Exacerbated I/R-Associated Heart Injury

**Figure 2:**
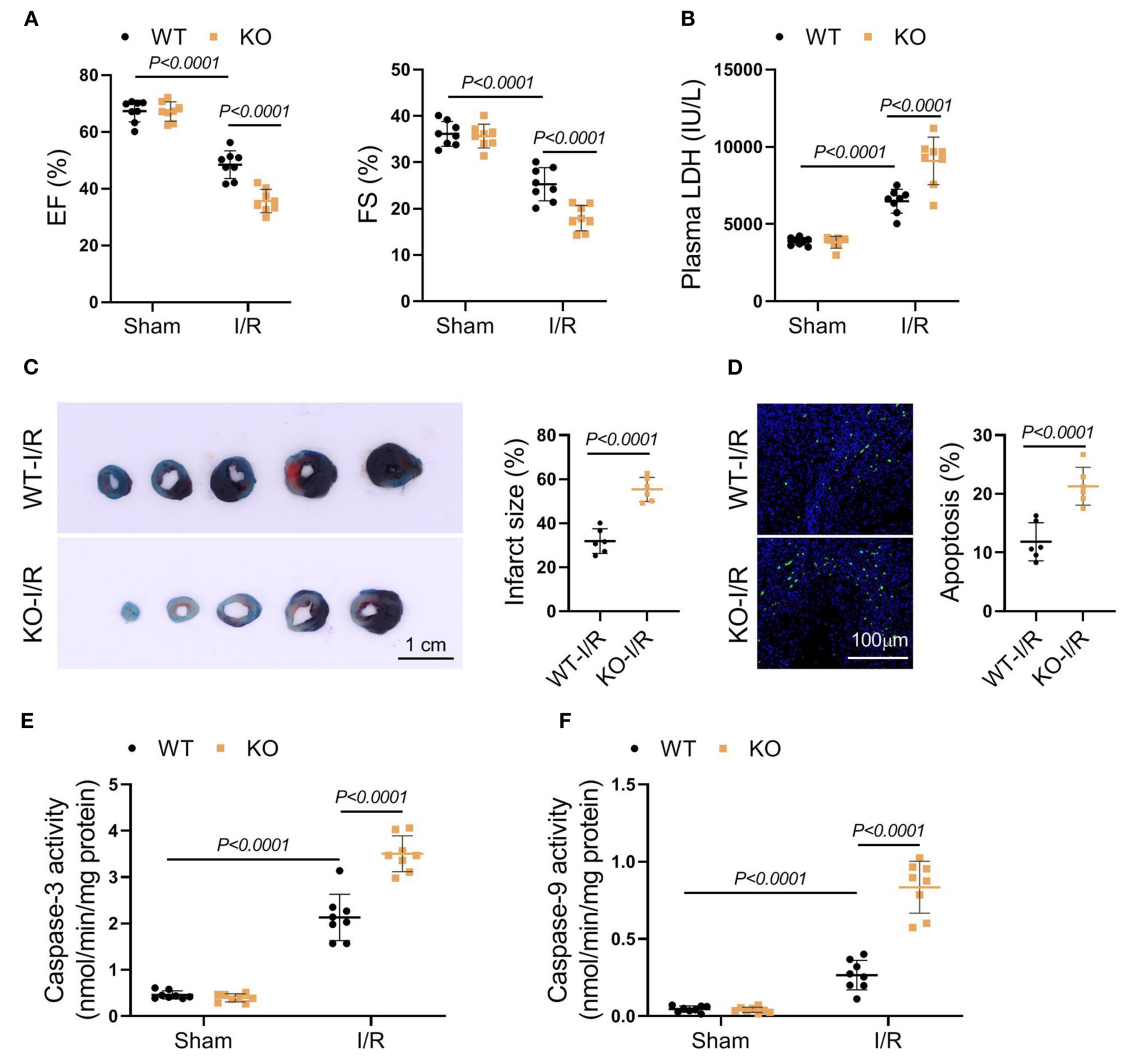
Genetic FASTK ablation exacerbated I/R injury. WT or FASTK KO mice were randomized to receive sham or I/R operation. **(A)** EF and FS values were calculated by echocardiography. **(B)** Plasma LDH levels were measured. **(C)** Myocardial infarct size was evaluated by TTC/Evens blue staining. **(D)** Cardiomyocyte apoptosis was determined by TUNEL staining. **(E)** Myocardial caspase-3 activity was measured and normalized to protein concentrations. **(F)** Myocardial caspase-9 activity was measured and normalized to protein concentrations. Data are shown as mean ± SD and analyzed by two-way ANOVA, followed by a Turkey test. WT, wild type; KO, knockout; EF, ejection fraction; FS, fraction shortening; LDH, lactate dehydrogenase; TTC, triphenyltetrazolium chloride; TUNEL, TdT-mediated dUTP Nick-End Labeling.

To clarify the role of FASTK in myocardial I/R injury, we induced myocardial I/R model in both WT and FASTK KO mice. Echocardiography showed that, compared with WT group, FASTK deficiency aggravated post-I/R cardiac contractile dysfunction as evidenced by much lower EF and FS values (Figure 2A). The release of LDH into the circulation post-I/R was also higher in KO mice when compared with WT group, suggesting that KO increased I/R-induced cardiac necrosis (Figure 2B). Structural analysis directly confirmed that genetic ablation of FASTK increased I/R-induced infarct size and cardiomyocyte apoptosis (Figures 2C,D). The activity of caspase-3 and caspase-9, two essential molecules initiating cardiomyocyte apoptosis, was also higher in the KO heart following I/R than those of the WT heart (Figures 2E,F). Taken together, these results provide solid evidence demonstrating that the down-regulation of FASTK is detrimental to the heart in the I/R process.

### Genetic Ablation of FASTK Aggravated I/R-Induced Mitochondrial Complex I Inactivation

**Figure 3:**
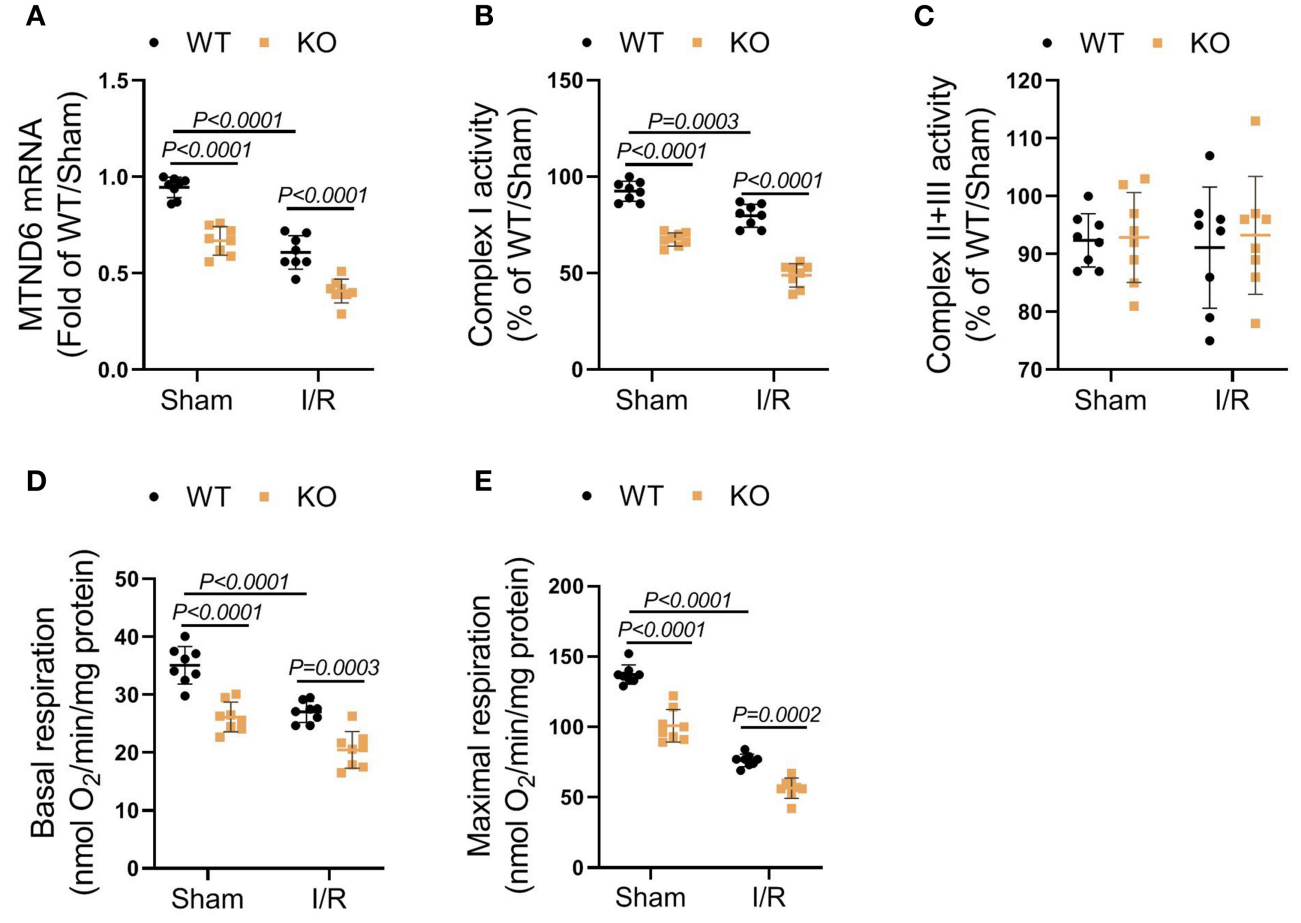
Genetic loss of FASTK exacerbated I/R-induced myocardial complex I dysfunction. Left ventricular tissues were collected from WT and FASTK KO mice who received sham or I/R operation. **(A)** MTND6 mRNA levels were determined by RT-PCR and normalized to β-actin mRNA expression. **(B)** Fresh cardiac mitochondria were collected and the complex I activity was measured. **(C)** The enzymatic activity of complex II plus III was measured in cardiac mitochondria isolated from WT and KO mice received sham or I/R operation. **(D,E)** Fresh heart mitochondria were collected and treated with complex I substrates (pyruvate and malate). Basal and ADP-stimulated maximal mitochondrial oxygen consumption rates were measured. Data are shown as mean ± SD and two-way ANOVA, followed by a Turkey test. ADP, adenosine diphosphate.

FASTK is recognized as a key modulator of mitochondrial complex I activity by specifically promoting MTND6 mRNA maturation (Jourdain et al., 2015). As expected, I/R obviously suppressed MTND6 mRNA expression and reduced mitochondrial complex I activity (Figures 3A,B). The suppressive effects of I/R on MTND6 expression and complex I activity were further augmented in the KO heart when compared with the WT group (Figures 3A,B). The activity of mitochondrial complex II plus III was unchanged after I/R in both WT and KO hearts (Figure 3C). Moreover, the functional analysis of fresh-isolated mitochondria showed that I/R suppressed complex I-supported mitochondrial respiration, no matter with or without adenosine diphosphate (ADP) stimulation (Figures 3D,E). In the KO heart, the suppressive effects of MI/R on mitochondrial complex I-mediated respiration were markedly exacerbated (Figures 3D,E). Collectively, these data identify that FASTK critically modulates myocardial MTND6 mRNA expression and mitochondrial complex I activity/respiration in the context of I/R.

### Replenishment of FASTK Expression Ameliorated sI/R-Induced Myocyte Death and Complex I Dysfunction

**Figure 4:**
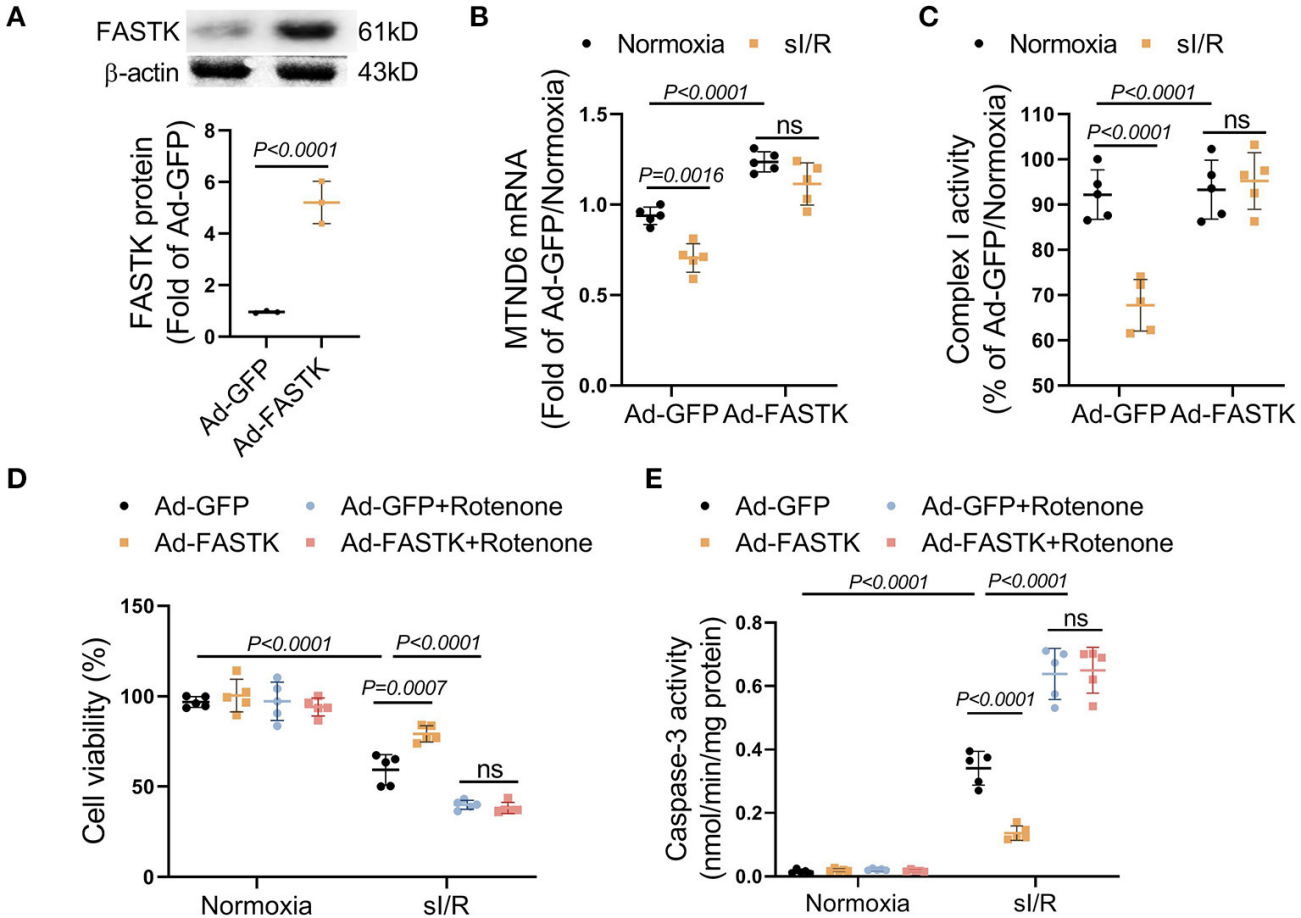
Replenishment of FASTK expression protected against sI/R injury through preserving complex I function. NMVMs were isolated WT mice and were transfected with adenovirus carrying GFP (Ad-GFP) or full-length FASTK (Ad-FASTK), followed by the exposure to normoxia or sI/R. **(A)** The protein levels of FASTK were determined by Western blot and normalized to β-actin expression. **(B)** MTND6 mRNA levels were determined by RT-PCR and normalized to β-actin mRNA expression. **(C)** The complex I activity was determined and normalized to intracellular protein abundance. **(D)** Ad-GFP or Ad-FASTK transfected NMVMs were exposed to normoxia or sI/R, co-treated with rotenone (0.1 μM). Cell viability was determined by MTT assay. **(E)** Caspase-3 activity was determined and normalized to intracellular protein concentrations. Data are shown as mean ± SD and analyzed by two-way ANOVA, followed by a Turkey test. sI/R, simulated ischemia/reperfusion; GFP, green fluorescent protein; MTT, 3-(4,5-dimethylthiazol-2-yl)-2,5-diphenyltetrazolium bromide.

We next explored the therapeutic potential of FASTK replenishment on MI/R injury. FASTK was overexpressed via adenovirus-mediated gene delivery in NMVMs (Figure 4A). Overexpression of FASTK ameliorated sI/R-induced MTND6 down-regulation and mitochondrial complex I inactivation in NMVMs (Figures 4B,C). Overexpression of FASTK markedly ameliorated sI/R-induced myocyte death and caspase-3 activation. Notably, the protective effects mediated by FASTK replenishment were totally abolished by the co-treatment with rotenone, a specific inhibitor of mitochondrial complex I (Figures 4D,E). Together, these *in vitro* data demonstrate that replenishment of FASTK expression is an effective strategy to ameliorate I/R injury by preserving mitochondrial complex I activity. Although rotenone itself was non-toxic to cardiomyocytes, sI/R-induced cell death was markedly aggravated by co-treatment with rotenone (Figures 4D,E). Above-mentioned results highlight that mitochondrial complex I is essential for cardiomyocytes to resist sI/R injury.

### Replenishment of FASTK Expression Ameliorated I/R-Associated Cardiac Injury and Complex I Dysfunction

**Figure 5:**
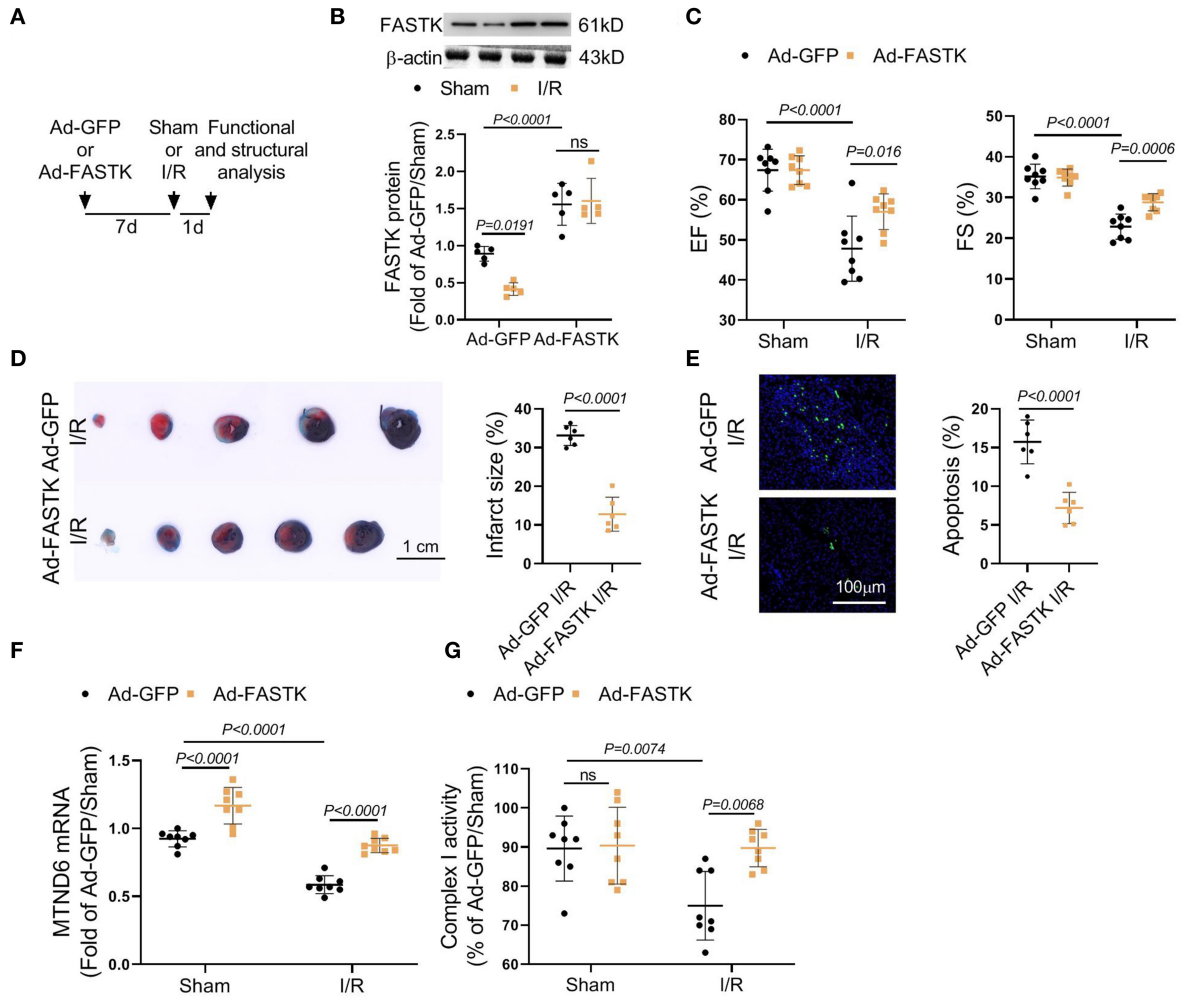
Overexpression of FASTK expression ameliorated I/R-associated cardiac injury and mitochondrial complex I inactivation. **(A)** Ad-GFP or Ad-FASTK vectors were delivered into WT mice by intra-myocardial injection. Seven days post-injection, mice were randomized to receive sham or I/R operation. One day post-operation, cardiac function and structure were analyzed. **(B)** Cardiac FASTK protein was determined by Western blot and normalized to β-actin expression. **(C)** EF and FS values were measured by echocardiography. **(D)** Myocardial infarct size was calculated by TTC/Evens blue staining. **(E)** Cardiomyocyte apoptosis was assessed by TUNEL staining. **(F)** Cardiac MTND6 mRNA levels were determined by RT-PCR and normalized to β-actin expression. **(G)** Complex I activity was measured in freshly isolated cardiac mitochondria. Data are shown as mean ± SD and analyzed by two-way ANOVA, followed by a Turkey test.

We next explored the therapeutic potential of FASTK overexpression on myocardial I/R injury by *in vivo* models. Adenovirus vectors carrying FASTK gene were delivered into the heart via intra-myocardial injection. Seven days after the injection, mice were randomly subjected to sham or I/R models (Figure 5A). Western blot confirmed that FASTK was overexpressed via intra-cardiac adenovirus injection (Figure 5B). Echocardiographic parameters showed that overexpression of FASTK ameliorated I/R-induced ventricular pump dysfunction as indicated by increased EF and FS values (Figure 5C). TTC/Evens blue staining showed that FASTK replenishment obviously reduced I/R-related myocardial infarction (Figure 5D). Consistently, FASTK overexpression also decreased cardiomyocyte apoptosis induced by I/R (Figure 5E). Mechanistically, FASTK overexpression preserved MTND6 mRNA levels and mitochondrial complex I activity during I/R process (Figures 5F,G). These *in vivo* data confirm that replenishment of FASTK expression is an effective strategy to preserve mitochondrial complex I functional integrity and protect against myocardial I/R injury.

## Discussion

In the present study, we have made several important observations. In response to the I/R insult, the reperfusion process decreases FASTK expression through bursting ROS production. Utilizing FASTK deficient mouse models, we confirm that genetic loss of FASTK exacerbates I/R-associated cardiac injury and mitochondrial complex I inactivation. Moreover, we observe that adenovirus-mediated FASTK overexpression preserves mitochondrial complex I function and ameliorates cardiac damage induced by the I/R injury. These data for the first time reveal that FASTK is an important molecule responsible for maintaining cardiac mitochondrial complex I functional integrity in the context of I/R injury.

Firstly, we find that FASTK expression is robustly repressed by the reperfusion process after the ischemic insult. FASTK is a protein localized in the mitochondrion and is highly expressed in tissues containing abundant mitochondria, such as the heart (Li et al., 2004). However, the role of FASTK in the regulation of cardiovascular physiology and pathophysiology is totally unknown. Utilizing myocardial I/R models, we show that cardiac FASTK expression is markedly inhibited by the reperfusion but not the myocardial ischemia. The revascularization of the ischemic myocardium often leads to an explosive production of ROS (Cadenas, 2018). We discover that the elimination of ROS reverses I/R-induced FASTK down-regulation. These results for the first time highlights an essential role of redox homeostasis in the modulation of FASTK expression.

Secondly, we confirm that the down-regulation of FASTK is a direct culprit to I/R-induced cardiomyocyte death. FASTK is initially known as a pro-survival molecule because it suppresses Fas activation-induced cell apoptosis (Tian et al., 1995). Given that cardiomyocyte apoptosis is a determinant of myocardial I/R injury, we speculate that FASTK might be involved in the regulation of cardiac I/R damage. Utilizing global FASTK knockout models, we for the first time assess the modulatory role of FASTK insufficiency in myocardial I/R injury. Consistently, genetic ablation of FASTK exacerbates I/R-induced infarction enlargement and cardiomyocyte loss. These *in vivo* observations are made in global FASTK deficient mice. There exists a limitation that the potential influence of FASTK loss in non-cardiac tissues on I/R damage could not be excluded. It is notable that FASTK overexpression ameliorated sI/R-induced cell death in cultured cardiomyocytes, revealing that a direct association between cardiac FASTK expression and I/R injury.

Thirdly, we recognize that FASTK is a key modulator of mitochondrial complex I function in the I/R process. The heart heavily relies on the energy substrate derived from the mitochondrial respiration (Maximilian Buja, 2017). However, mitochondrial complex I is highly susceptible to functional and structural destroy in response to I/R, thereby contributing to mitochondrial respiratory suppression, energy supply crisis, and eventually cardiomyocyte death (Chen et al., 2019; Galkin, 2019). However, the molecular mechanism underlying I/R-associated complex I dysfunction is largely unknown. FASTK is recently identified as a RNA-binding protein to promote MTND6 mRNA maturation (Jourdain et al., 2015). Genetic ablation of FASTK causes a marked reduction of complex I activity in mammalian cells, highlighting FASTK is essential to maintain normal complex I function (García Del Río et al., 2018; Gomez-Niño et al., 2018). The present study shows that the FASTK deficient heart is highly susceptible to I/R-induced complex I dysfunction and respiration, confirming that FASTK critically modulates myocardial mitochondrial complex I activity even under stressful conditions such as I/R. These results also provide a direct evidence demonstrating that the down-regulation of FASTK underlies I/R-induced complex I dysfunction.

Last but not the least, we, for the first time, clarify that the replenishment of FASTK expression is a promising therapeutic strategy for the prevention of myocardial I/R injury. Utilizing adenovirus-mediated gene delivery, we also test the therapeutic potential of FASTK overexpression. Adenovirus-mediated FASTK overexpression significantly protects the heart against I/R-associated mitochondrial complex I inactivation, cardiomyocyte loss, infarction enlargement, and pump dysfunction. These preclinical animal experiments show that FASTK might be an effective target to intervene myocardial I/R injury. Notably, the therapeutic effects of FASTK overexpression are totally abolished in cultured cardiomyocytes by the co-treatment of rotenone, a specific inhibitor of mitochondrial complex I. These data suggest that FASTK-mediated cardioprotection is achieved by the mitochondrial fashion.

Taken together, the present study for the first time demonstrates that the down-regulation of FASTK is a direct culprit of I/R-associated mitochondrial complex I dysfunction and cardiomyocyte death. Replenishment of FASTK expression is a promising and effective therapeutic strategy for the prevention of myocardial I/R injury.

## Data Availability Statement

The raw data supporting the conclusions of this article will be made available by the authors, without undue reservation.

## Ethics Statement

The animal study was reviewed and approved by Air Force Medical University Committee on Animal Care.

## Author Contributions

FZ and XC designed the study, gained the funding support, and revised the manuscript. XC, GH, and CL performed the experiments and analyzed the data. XC and YW drafted the manuscript. All authors contributed to the article and approved the submitted version.

## Conflict of Interest

The authors declare that the research was conducted in the absence of any commercial or financial relationships that could be construed as a potential conflict of interest.
